# Exploring the Effect of Assisted Repeated Reading on Incidental Vocabulary Learning and Vocabulary Learning Self-Efficacy in an EFL Context

**DOI:** 10.3389/fpsyg.2022.851812

**Published:** 2022-02-18

**Authors:** Habib Soleimani, Farnoosh Mohammaddokht, Jalil Fathi

**Affiliations:** Department of English and Linguistics, Faculty of Language and Literature, University of Kurdistan, Sanandaj, Iran

**Keywords:** repeated reading, assisted reading, incidental vocabulary learning, vocabulary learning self-efficacy, smartphone

## Abstract

The purpose of the current study was to investigate the effect of two types of repeated reading (i.e., assisted and unassisted) on incidental vocabulary learning of Iranian English as a Foreign Language (EFL) learners. In so doing, a sample of 45 intermediate EFL students from two intact classes of a language institute were selected as the participants. The two classes were randomly assigned to an unassisted group (*N* = 21) who were required to just *read* and an assisted group (*N* = 24) who were asked to *read* and *listen* to 24 short texts several times. The assisted group employed their smartphones to listen to the audio files of the short stories. The data were gathered *via* a researcher-made vocabulary test and vocabulary learning self-efficacy scale. The results of ANCOVA revealed that although both types of repeated reading contributed to enhancing vocabulary learning of the participants, assisted repeated reading led to significantly greater EFL vocabulary gains. Additionally, the findings revealed that both assisted and unassisted repeated reading improved vocabulary learning self-efficacy of the participants and there was not a significant difference between the two types of interventions. The findings of the present study have implications for EFL researchers and practitioners.

## Introduction

Vocabulary learning is long regarded as an essential aspect of second and foreign language (L2/FL) learning (Nation, 2013). Through vocabulary learning, L2 learners can achieve mastery over the second language (Nation, 2008). Since practitioners and scholars have come to realize the integral role of vocabulary learning in communication and language learning, more research attention has been given to L2 vocabulary instruction (Rassaei, 2017; Yousefi and Biria, 2018; Liu et al., 2020). L2 literature has observed a growing interest in exploring effective explicit and implicit strategies such as using dictionaries, inferring word meaning from context, and extensive reading for learning collocations and vocabulary in particular (see Hunt and Beglar, 2005). Research has also shown that a considerable amount of vocabulary is learned receptively through listening or reading (Nagy et al., 1985). In the meantime, due to growing popularity of new technologies and applications, numerous researchers and practitioners have investigated and employed mobile assisted language learning (MALL) as a viable technique for L2 vocabulary teaching and learning (Burston, 2013; Rassaei, 2018, 2020; Lin and Lin, 2019).

Given that reading has been considered as a critical source of vocabulary growth (Zahar et al., 2001), the role of reading for L2 vocabulary learning should receive further attention. What is commonly agreed upon is that some part of one’s L2 vocabulary is acquired incidentally through reading (Ramos and Dario, 2015). One of the most influential procedures within L2 education investigations is extensive reading (Nakanishi, 2015) which has been emphasized to develop vocabulary learning (Wang, 2013). Extensive reading is a type of reading that provides learners with exposure to large quantities of materials (Grabe and Stoller, 2002) for comprehension often without performing any tasks after reading. Extensive reading has been continuously reported as the most commonly recommended model improving learners’ language proficiency such as reading comprehension (Nakanishi, 2015), grammar knowledge (Ellis, 2005) and incidental vocabulary learning (Horst, 2005; Suk, 2017). Nonetheless, in order for the learning to occur, it is worth stressing that the reading materials need to be selected according to the learners’ language proficiency and reading abilities (Suk, 2017). When learners do multiple readings, they have rapid access to known words and word patterns in various contexts through encountering them repeatedly. Over the time, learners’ vocabulary size tends to develop and they can also achieve a deeper understanding of the new words. Words which are learned in this procedure can be incorporated into learners’ writing and speech (Nation, 2008).

Investigations into incidental vocabulary learning have revealed that the number of times a new and unknown word is repeated in a text influences how likely individuals will learn the word successfully (e.g., Peters and Webb, 2018). In this regard, delving deeply into vocabulary development, a number of empirical studies have been carried out to explore the effectiveness of repeated reading method in the past decades, hoping to find out how incidental vocabulary learning can occur through this approach (see Webb and Chang, 2012). In the repeated reading approach, the students read and reread a passage several times (two to four times) aloud (Samuels, 1979) or silently (Anderson, 1993) in a predetermined level of pace until reading the text fluently (Meyer and Felton, 1999). In this approach, learners read specified texts from graded readers (e.g., books and passages that have simplified grammatical structures and reduced vocabulary range) repeatedly in order to develop word recognition as well as improving reading fluency and comprehension (Dlugosz, 2000). There are two kinds of repeated reading: assisted and unassisted repeated reading procedures. Unassisted repeated reading is where students read short passages autonomously without any audiotape to follow until they reach a fluent reading state. With the same procedure, in assisted form of repeated reading students read along while listening to the audiotape or a live model (Webb and Chang, 2012). Samuels who first coined the term repeated reading pinpointed that unassisted repeated reading method increases poor readers’ comprehension and oral fluency (Samuels, 1976, 1979). In addition, Chomsky (1978), found that the repeated reading approach caused slow readers to become more motivated, confident, and willing to read new materials independently.

Repeated reading has been regarded as one of the most effective approaches for acquiring vocabulary because it can expose L2 students to massive amounts of meaningful input. Therefore, these multiple and consistent exposures and repetition contribute to the incidental acquisition of novel English vocabulary (Suk, 2017). With regard to the EFL context, some researchers have highlighted the effective role of assisted repeated reading in vocabulary gains (Webb and Chang, 2012; Liu and Todd, 2016; Serrano and Huang, 2018) and fluency development (Taguchi et al., 2004). According to Serrano and Huang (2018), students are able to learn vocabulary even when the assisted repeated reading focuses on comprehension. Furthermore, Taguchi et al. (2004) argued that assisted repeated reading has the potential to develop readers’ fluency as well as helping them become independent readers.

Concerning self-efficacy, Bandura (1977, 1978) believed that individuals with high assurance in their skills and capabilities can succeed in performing a difficult task and see it as a challenge to be mastered not as a threat to be avoided. One’s self-efficacy determines not only their persistence, endeavor, and strategizing, but their subsequent job performance (Heslin and Klehe, 2006). Some educators so far have adapted self-efficacy and categorized it further, for instance, into professional self-efficacy, multitasking self-efficacy, and computer self-efficacy, and (Islam et al., 2018). Among other dimensions of self-efficacy, in this study we decomposed self-efficacy into vocabulary self-efficacy. As far as EFL context is concerned, skill-specific self-efficacy has recently received some attention in empirical studies (e.g., Fathi et al., 2019, 2020; Fathi and Soleimani, 2020; Rahimi and Fathi, 2021).

Although there is a plethora of studies investigating vocabulary learning through reading as well as the impressive progress which has been made by L2 researchers (see Horst, 2005; Webb, 2007; Webb and Chang, 2012), some gaps still exist. First, research lacks a comprehensive and conclusive theory of in what ways incidental vocabulary is learned through repeated reading in an EFL context. Although Webb and Chang (2012) and Serrano and Huang (2018) underscored the effectiveness of assisted and unassisted repeated reading in vocabulary learning among Taiwanese EFL learners, further studies should be carried out in EFL contexts. Second, repeated reading (i.e., assisted and unassisted) research provides relatively little evidence about the incidental vocabulary-expanding impacts of reading repeatedly, simply because L2 literature mostly tends to focus on more general aspects of language development. Furthermore, given the widespread recognition of MALL (Burston, 2013), the use of smartphones for assisted repeated reading has remained under-researched. Finally, to the best of our knowledge, no study to date has explored the effect of repeated reading, including its both forms on vocabulary learning self-efficacy among EFL learners. The rationale behind investigating vocabulary learning self-efficacy was the fact that self-efficacy is argued to influence vocabulary learning and the use of appropriate strategies for vocabulary learning (Mizumoto, 2012, 2013; Hong et al., 2014). In addition, although L2 vocabulary has received significant research attention, self-efficacy in vocabulary learning has remained under-explored (Mizumoto, 2013; Nation, 2013). Given the insufficient available data, further investigation is required to determine whether and exactly how repeated reading leads to vocabulary learning. In an attempt to bridge the gap in the current literature, this study investigated the scope and depth of incidental vocabulary learning and vocabulary learning self-efficacy through assisted and unassisted repeated reading in the EFL setting of Iran. Therefore, the following research questions guided this research:

1. Does assisted repeated reading have any significant effect on incidental vocabulary learning of EFL learners?
2. Does assisted repeated reading have any significant effect on vocabulary learning self-efficacy of EFL learners?

## Review of Literature

It is commonly thought that vocabulary learning is pivotal in L2/FL learning (e.g., Nation, 2013). Language learners might face an immense vocabulary challenge since they require to learn different dimensions of each word, including its aural and textual forms, collocations and associations (Nation, 2013), in order to successfully understand and use L2 communicatively. Researchers have also stressed the significance of word knowledge in reading comprehension (see Read, 2000). It has been argued that a learner who knows more than 98% of the words used in a passage can fully understand it autonomously (e.g., Schmitt et al., 2011). Therefore, it can be argued that a good vocabulary knowledge including both its breadth and depth is a significant element of reading competencies.

Cronbach’s (1942) conceptualization categorized vocabulary knowledge into two major divisions: first, knowledge of word meaning (generalization, precision and breadth of meaning) and second, degrees of accessibility to this knowledge (application and availability). Later, Richards (1976) introduced other factors such as syntax, register, frequency, association, derivation, polysemy, and semantic features involved in knowing a word. Qian (1999) also proposed two key aspects of vocabulary knowledge: Vocabulary breadth knowledge that is related to the size of vocabulary and depth of vocabulary knowledge that is referred to how deep and well one knows a word.

The extant literature on L2 learning provides much empirical credit to the significance of vocabulary in the language acquisition process (Read, 1988). To date, some topics related to vocabulary in EFL/L2 have been investigated. In particular, the vocabulary knowledge of EFL learners has attracted increasing attention (Rassaei, 2017; Yousefi and Biria, 2018). Some studies have examined EFL learners’ vocabulary learning through explicit instruction (Mizumoto and Takeuchi, 2009), computer-assisted language learning (Shokrpour et al., 2019; Namaziandost et al., 2021), extensive reading (Liu and Zhang, 2018; Song, 2020), and repeated reading (Liu and Todd, 2016). Considering the fact that vocabulary learning process is complex and explicit vocabulary teaching in EFL classrooms could only cover a small proportion of new words that students learn, it is important to find other ways for teaching vocabulary. What can be found in the current literature is some mentions of instructional techniques for enhancing L2 vocabulary learning reported by different instructors, such as the learning of word lists (Carter, 1987), learning words in a discourse context (Laufer, 2003), inferencing (Alahmadi and Foltz, 2020), exposure to word glosses (Webb, 2007), songs (Pavia et al., 2019), games or stories (Chou, 2014). The detailed accounts of the implementation of these techniques in real EFL classrooms and their effectiveness in vocabulary teaching have been the concern of many researchers. Teaching vocabulary seems laborious for EFL teachers since teaching English is very likely to encounter many obstacles and challenges. As Gorsuch et al. (2015) mentioned, one challenge for FL programs is to provide students with sufficient input and experience to use the language. There are few opportunities for EFL learners to use English in the real world since they have little or even no exposure to the language beyond the classroom walls (Read, 1988).

Scholars and practitioners have become growingly cognizant of the salient effect of reading, more particularly extensive reading on vocabulary knowledge (Horst, 2005). Extensive reading refers to a kind of L2 reading approach in which (a) learners are provided with a considerable number of reading materials with the purpose of reading for pleasure; (b) they read their selection at a fast rate to obtain a general understanding of the text; (c) they are more related with comprehending the whole passage rather than individual sentences or words (Day et al., 1998). Intensive reading, in contrast, often is concerned with the careful and precise reading of more difficult, shorter FL passages with the purpose of detailed and thorough comprehension under the guidance of the teacher (Carrell and Carson, 1997).

Nation and Wang (1999) asserted that graded readers can be seen as one of the key sources of vocabulary learning for L2 learners if used appropriately. As extensive reading approach provides students with the chance to face different vocabularies in their context of use, it can be very pleasant and motivating which in turn can facilitate learner autonomy (Thornbury, 2002). A bulk of L1 and L2/FL reading research has mentioned the potential merits of extensive reading in expanding language learners’ vocabulary learning (Pigada and Schmitt, 2006; Liu and Wu, 2011). One example of such investigations is Pigada and Schmitt’s (2006) study which investigated the effect of extensive reading on vocabulary. The study tested 133 words in a month with a learner of French as a FL. The results showed that nearly two-thirds of the target words were learned. This is to say, extensive reading can be an effective approach in promoting incidental vocabulary learning. In a subsequent study, Liu and Zhang (2018) did a meta-analysis study in this field of research. Their first goal was to discover the role of extensive reading in vocabulary learning. The second aim was to find appropriate teaching methods and teaching length in order to have an effective extensive reading program. The findings of their study indicated that extensive reading led to significantly greater vocabulary knowledge (English vocabulary). They also suggested that extensive reading can develop learners’ vocabulary in one semester (less than 3 months). In addition, different comprehension questions, vocabulary exercises, and graded readers are appropriate and influential teaching methods and reading materials for improving EFL learners’ vocabulary promoting.

In a similar vein, the results from Kweon and Kim’s (2008) study demonstrated that the participants achieved vocabulary gains after the EFL extensive reading intervention. It was also revealed that the participants found nouns easier to retain compared to verbs and adjectives. Furthermore, they learned frequent words more easily than less frequent words. Moreover, Horst’s (2005) pilot study indicated that the participants learned mostly half of the unfamiliar words they faced in the extensive reading materials they selected. In this line of research, Wang (2013) made an attempt to examine the effect of extensive reading on the word knowledge of EFL Taiwanese learners. This was a 15-week extensive reading procedure in which the participants were required to read 30 English texts in this period. The findings underscored the significant impact of extensive reading on English vocabulary growth. It was claimed that the EFL extensive reading program plays a beneficial role in incidental vocabulary learning among EFL learners with lower competence. However, implementing extensive reading without the supervision of the teacher outside the classroom may lead students to face difficulties (Martina et al., 2020).

In the last few years, L2 researchers and scholars have assembled an intriguing list of research probing repeated reading (several readings of a passage) as an approach for enhancing reading competencies (e.g., Gorsuch and Taguchi, 2010). Although there is still much to be learned about this procedure, evidence shows that repeated reading is a viable instructional model for both disabled and developmental readers (Therrien, 2004).

Rereading the same text using either the assisted or unassisted repeated reading procedure significantly enhances reading rate and accuracy (Chomsky, 1978; Samuels, 1979). In repeated reading students read a meaningful passage repeatedly until oral production is flowing and fluid and results in increased comprehension and fluency (Taguchi, 1997). Basically, repeated reading falls into two categories: assisted repeated reading (*read-along*), in which a student reads the passage while audiotaped or live model of the text is used (Chomsky, 1978). And unassisted repeated reading (*independent practice*), where the student reads the passage autonomously while no model or prototype is used (Samuels, 1979). Samuels (1979) devised the repeated reading approach as means of supporting incompetent readers achieve automatic word recognition. In this procedure, students were required to reread a meaningful passage aloud until reaching a criterion degree of fluency.

Repeated reading has so far drawn increasing attention from researchers as a potentially effective method for reading and comprehension among L1 (Kuhn and Stahl, 2003) and L2 readers (Webb and Chang, 2012; Gorsuch et al., 2015). According to Blum and Koskinen (1991) the positive effect of repeated reading on reading fluency has been revealed to have other considerable benefits for learners. They maintained that repeated reading not only improves reading comprehension and fluency, but also helps learners become more motivated to read and more confident in their reading.

Repeated reading is also a method which scaffolds students with reading disabilities to build fluency (Therrien and Hughes, 2008; Lee and Yoon, 2017). For example, students with reading disabilities in the study carried out by Lee and Yoon (2017) showed high reading fluency after the repeated reading intervention. They also stressed the effectiveness of the combination of repeated reading and a listening passage preview for students with reading disabilities, more specifically for those at the elementary level. Furthermore, Therrien (2004) found that repeated reading is an effective procedure for both students with learning disabilities and nondisabled students by which their reading fluency and comprehension increases.

The related literature evinces that audio-assisted repeated reading is a promising procedure for improving L2 readers’ fluency (Taguchi et al., 2004). Repeated reading of meaningful passage, including listening-while reading (assisted form) has been found to produce improvements in reading fluency, rate and word recognition accuracy (Rasinski, 1990). The findings from Taguchi et al.’s (2004) study yielded that repeated reading helped students to learn and retain vocabulary and grammar. Students were also able to monitor their reading comprehension through multiple readings. It was also revealed that neither successive re-readings nor audio-models were tedious and distracting for a considerable number of participants.

As far as L2 vocabulary learning is considered, many scholars have corroborated the effectiveness of repeated reading for vocabulary growth (Serrano and Huang, 2018). Drawing on insights acquired from investigations into repeated reading as well as on reading aloud, some researchers believe that repeated encounters with new forms in various contexts make repeated reading instrumental for incidental L2 vocabulary learning (Brown et al., 2008). Incidental vocabulary learning research has verified the assumption that much exposure to L2 reading texts can contribute to vocabulary growth (Webb and Chang, 2012).

In this line of research, several studies have explored the incidental vocabulary learning through repeated reading (Brown et al., 2008; Webb and Chang, 2012; Liu and Todd, 2016; Serrano and Huang, 2018). For instance, the main focus of the study conducted by Webb and Chang (2012) was on the influence of repeated reading on incidental vocabulary learning. The findings revealed that both kinds of repeated reading (assisted and unassisted) contributed to improvement in incidental vocabulary learning. More precisely, the Taiwanese learner of English as a FL reported significant word gains through assisted repeated reading.

Similarly, Serrano and Huang (2018) explored the impact of assisted form of repeated reading on vocabulary learning. Taiwanese EFL students as participants of this study were divided into two groups of intensive and spaced. The participants in one group were provided with an assisted repeated reading program (reading while listening) once every day (intensive) while the learners in the other were supposed to read the same passage once every week (spaced). The results showed that vocabulary gains were achieved after the assisted repeated reading treatment. It is worth mentioning that the participants in the intensive group exhibited significantly greater vocabulary achievement compared to the spaced group. This suggests that concentrated practice contributes to greater vocabulary learning.

More recently, Serrano and Huang (2018) set out a partial replication study of Serrano and Huang’s (2018) previous research into incidental vocabulary learning. This study used the same context, methodology, design, and analyses as the original study; the level of the target vocabulary learning was the only different factor. The results showed a significant intentional vocabulary gains after the repeated reading interval. The only common finding between two studies refers to immediate vocabulary gains. However, Liu and Todd (2016) argued that repeated reading seems more effective with target words that have etymological roots with the learners’ L1.

In recent years, the role of non-cognitive factors of language learning, such as self-efficacy motivation, and emotions has come under the spotlight. In a general sense, self-efficacy refers to individuals’ perceptions towards their ability in accomplishing a specific task successfully (Bandura, 2011; Fathi et al., 2021). The important non-cognitive trait influences achievement, skills, knowledge, perceived value, and outcome expectations (Schunk, 2003). As Linnenbrink and Pintrich (2003) argued, efficacious learners are more likely to persist, seek help, and work hard, so they can successfully complete a task. Extending this concept into the domain of reading, self-efficacy is the reader’s belief about their capability to read effectively (Guthrie and Wigfield, 1999). According to Wigfield et al. (2004), self-efficacious readers have not only better performance but also tend to persist through difficult reading tasks.

The focus of the current study is the effect of repeated reading on learners’ self-efficacy in learning words, vocabulary self-efficacy more specifically. Less is known about the association between self-efficacy and repeated reading, because previous studies have mainly focused on the effect of repeated reading on reading comprehension. To the best knowledge of researchers, no study to date has investigated the impact of repeated reading on vocabulary self-efficacy in the EFL context.

In summary, the review of the existing literature indicated that incidental vocabulary learning through repeated reading (i.e., assisted and unassisted) has not been seriously taken into consideration by the practitioners in the domain of EFL, in the context of Iran in particular. Unfortunately, no studies so far have systematically and explicitly investigated incidental English vocabulary learning in Iran. The data regarding vocabulary and vocabulary mastery in EFL, however, is not sufficient to give a vivid picture of the role of repeated reading since vocabulary growth seems to be neglected. Therefore, little information is available with regard to the rationale behind EFL instructors’ adoption of repeated reading approach for incidental vocabulary learning. In order to shed more light on this procedure, the current study examined the effect of assisted and unassisted repeated reading on incidental vocabulary learning and sought to determine whether and to what extent lexical knowledge of the participants was developed.

## Materials and Methods

### Participants

A total number of 45 Iranian EFL students took part in this quasi-experimental study. These participants were pre-intermediate students of two intact groups in a private language institute in Tehran, Iran. The two classes were randomly assigned to an assisted repeated group (*N* = 21) and an unassisted repeated group (*N* = 24). All the participants were female students ranging in age from 13 to 16, with the mean age of 14.56. Also, they had the experience of at least 4 to 6 years of learning English as a foreign language either in public schools or in the private language teaching institutes. The global English proficiency of the participants was measured by a version of Preliminary English Test (PET). The mean scores of PET were compared by running an independent samples *t*-test whose results indicated that there was no statistically significant difference between the groups.

### Reading Materials

The reading materials were 26 short stories selected from http://eslyes.com/eslread/. Much care was exercised to choose stories of similar level of difficulties. Table 1 presents selected short stories and their linguistics features. As seen in the table, Flesch–Kincaid Grade Level of all stories ranged from 3 to 3.6. Their ease score varied from 85.9 to 93.1. Most texts lacked passive sentences and the texts had from minimum of 8.6 to maximum of 12.1 average number of words per sentence. The total number of words varied from 192 to 320. In addition to the selected 26 short stories, the students were required to study *Top Notch 2*, a famous commercially published series, as the major textbook prescribed by the institute.

**Table 1:** Selected short stories and their linguistics features.

	Short story title	Flesch–Kincaid grade level	Flesch reading ease score	%Passive sentences	Average number of words per sentence	Total number of words
1	Man flies 200 miles in chair	3.0	93.1	0%	11.3	226
2	Cleaning a dirty plate—oops!	3.4	88.7	0%	10.5	316
3	Toilet tank almost overfills	3.4	87.7	0%	9.7	282
4	A play, or a movie? neither!	3.2	91.6	4%	11.7	247
5	You’re not my dad	3.5	88.2	0%	10.8	305
6	A haircut every 2 weeks	3.3	92.5	0%	12.1	219
7	Yardman mows and blows	3.3	91.4	0%	12.1	209
8	Finds bargains at thrift shop	3.3	89.4	4%	10.3	227
9	Pete’s too sharp knife	3.2	87.7	4%	8.9	223
10	A good hot dog sandwich	3.5	87.5	0%	10.0	211
11	Loose button gets sewn	3.5	88.8	4%	10.7	225
12	Paper pile grows and goes	3.1	90.3	0%	10.1	192
13	A daytime robbery in LA	3.5	86.0	0%	9.3	214
14	English is so hard	3.2	90.4	0%	11.1	201
15	Let us buy some paint	3.5	86.9	0%	9.7	283
16	A big cash wedding gift	3.3	88.7	3%	10.0	291
17	New to America from Asia	3.2	87.0	0%	8.6	199
18	Am i having a heart attack?	3.4	87.3	3%	10.0	320
19	Scrooge brings christmas gift	3.3	88.0	0%	11.1	211
20	His haircut leaves her cold	3.2	88.7	0%	9.5	209
21	Driving lesson scares them both	3.4	87.0	0%	9.2	304
22	God: open door, feed flies	3.3	90.8	0%	11.5	230
23	He: fraud; She: little white lie	3.2	89.0	0%	10.2	316
24	Man hoards library books	3.4	89.1	3%	10.6	278
25	TSA revises jewelry regs	3.4	85.9	13%	9.5	219
26	Cancer? white spot on tongue	3.6	86.8	6%	10.0	312

### Materials and Instruments

#### Preliminary English Test

Before beginning the treatment, the homogeneity of the students concerning global English proficiency was checked. To this end, a version of Preliminary English Test (PET) by Cambridge English for Speakers of Other Languages (ESOL, 2009) was given to the students of both groups. This PET version comprised three components including Reading (5 sections), Listening (4 sections), and Speaking (4 sections). The reliability coefficients of the reading and listening components were 0.83 and 0.79, respectively. Also, the inter-rater reliability coefficient for the speaking component was reported to be 0.81.

#### Vocabulary Test

Vocabulary learning of the participants was assessed by a 70-item multiple choice test designed by the researchers. The items of this test were randomly chosen from the shorts stories which were provided to both groups. The stems of the items were selected from the statements of the stories and the distractors were also the vocabularies included in content of the short stories. Two parallel forms of the test were designed for the pre-test and post-test by altering the order of items and distractors. The face and content validity of the test were approved by three domain experts. In addition, a pilot study was performed to gain preliminary information regarding the reliability and appropriateness of the items. The internal consistency of the test, as estimated by KR-21, was 0.79.

#### Vocabulary Learning Self-Efficacy Scale

Vocabulary learning self-efficacy of the participants was measured by the 4 items adapted from Mizumoto (2013). Each item was assessed on a 6-point scale varying from 1 (not at all true of me) to 6 (very true of me). A sample item of the scale is “I am good at memorizing vocabulary.” The reliability estimate of this scale, as calculated by Cronbach’s Alpha formula, was reported to be 0.78 in this study.

### Procedure

This experiment began in summer semester of 2019. During the first session, the researcher administered the PET as well as the pre-tests of the study which included the vocabulary test and VLSS. The purpose of this course was to improve the general language proficiency of the participants. However, in addition to *Top Notch 2*, the students of both groups were provided with short stories as the supplementary materials. The two groups were taught by the same instructor at a private language institute. Following the procedure used by Webb and Chang (2012), the researchers began the study intervention. The intervention which lasted for 13 sessions was carried out to investigate the effects of repeated reading of 26 short stories over a 13 session period. Both groups were required to read two short stories in each session. More specifically, the unassisted group students were required just to read the stories, whereas the students of assisted group were required to read and listen to the stories. The assisted group students were also provided with audio files of the stories in addition to the texts. The assisted group employed their smartphones to listen to the audio files of the short stories. However, the students of the unassisted group were just provided with the printed version of stories without audio files and they were not required to use their smartphones as the course requirement. The students of both groups were required to either read (i.e., unassisted) or read and listen (i.e., assisted) to each story at least three times. The purpose of both reading conditions was comprehension of the texts without any focus on any particular grammatical structure or vocabulary explanation. The students could make use of dictionary, discuss the texts with each other, or ask questions. At the end of the treatment, the post-tests (vocabulary test and VLSS) were given to the participants of both groups.

### Data Analysis

Both descriptive and inferential statistics were employed for the data analysis. Concerning the inferential data analysis, the collected data were analyzed by performing one-way between-groups analyses of covariance (ANCOVA). The purpose of running ANCOVA was to compare the impacts of the two types of reading instructions (i.e., assisted vs. unassisted) used in the two groups on vocabulary learning and vocabulary learning self-efficacy as the two dependent variables of the study.

## Results

To ensure the homogeneity of the participants in terms of global English skills, an independent-samples *t*-test was performed to compare the PET scores for the assisted and unassisted groups. As observed in Table 2, the outcomes showed that no significant difference was observed in the PET scores for the assisted group (*M* = 25.16, SD = 8.28) and the unassisted group [*M* = 27.08, SD = 9.01; *t*(43) = −0.621, *p* > 0.05], confirming the fact that both groups were homogeneous before beginning the intervention.

**Table 2:** Results of the PET for each group.

Groups	*M* (SD)	*T*	Sig.
Assisted group	25.16 (8.28)	−0.621	0.436
Unassisted group	27.08 (9.01)		

Afterwards, in order to explore the impacts of the two types of repeated reading on the participants’ vocabulary learning and vocabulary learning self-efficacy, a number of one-way between-groups analysis of covariance (ANCOVA) were conducted to compare the effects of the two types of L2 reading instructions (i.e., assisted vs. unassisted repeated reading) used in the two groups on the two dependent variables.

Concerning the impact of using the repeated reading on EFL learners’ vocabulary learning, as indicated in Table 3, the vocabulary learning mean score of the assisted group was 65.33 (SD = 11.85) on the pre-test and it was increased to 81.04 (SD = 12.84) on the post-test. By the same token, the mean score of vocabulary learning on the pre-test for the unassisted group was raised from 64.12 (SD = 10.32) to 72.20 (SD = 11.49) on the post-test. However, after adjusting for the pre-test scores of vocabulary learning, it was revealed that there was a statistically significant difference between the two groups on post-test scores of vocabulary learning, [*F*(1, 42) = 7.97, *p* = 0.007, partial *η*^2^ = 0.16; see Table 4]. This finding demonstrated that the participants of the assisted group improved their vocabulary learning significantly more than the participants of the unassisted group, demonstrating that the assisted repeated reading instruction was significantly more effective in enhancing the L2 vocabulary learning of the participants.

**Table 3:** Descriptive statistics for pre- and post-tests scores.

Groups	Scales	Pre-test		Post-test	
		*M*	SD	*M*	SD
Assisted group	Vocabulary	65.33	11.85	81.04	12.84
	Self-efficacy	2.64	0.54	3.07	0.59
Unassisted group	Vocabulary	64.12	10.32	72.20	11.49
	Self-efficacy	2.77	0.58	3.27	0.47

**Table 4:** The results of ANCOVA on vocabulary size.

Source	Type III sum of squares	df	Mean square	*F*	Sig.	Partial *η*^2^
Corrected model	3460.320	2	1730.160	19.348	0.000	0.480
Intercept	1193.185	1	1193.185	13.343	0.001	0.241
Pre.vocabulary	2585.230	1	2585.230	28.911	0.000	0.408
Group	713.199	1	713.199	7.976	0.007	0.160
Error	3755.680	42	89.421			
Total	269421.000	45				
Corrected total	7216.000	44				

With regard to vocabulary learning self-efficacy, the descriptive statistics data (see Table 3) indicate that the mean score of the vocabulary learning self-efficacy for the unassisted group was 2.77 (SD = 0.58) in the pre-test and it was raised to 3.27 (SD = 0.47) on the post-test. Similarly, the vocabulary learning self-efficacy mean score for the assisted group was 2.64 (SD = 0.54) on the pre-test and this value was raised to 3.07 (SD = 0.59) on the post-test. After adjusting for the pre-test scores of vocabulary learning self-efficacy, the results of ANCOVA (see Table 5) showed that there was not any statistically significant difference between the two groups on post-test scores of vocabulary learning self-efficacy, [*F*(1, 42) = 1.04, *p* = 0.312, partial *η*^2^ = 0.02]. This finding revealed that both assisted and unassisted repeated reading instructions equally enhanced the vocabulary learning self-efficacy of the participants and there was not any significant difference between them.

**Table 5:** The results of ANCOVA on vocabulary learning self-efficacy.

Source	Type III sum of squares	df	Mean square	*F*	Sig.	Partial *η*^2^
Corrected model	7.519	2	3.759	30.719	0.000	0.594
Intercept	2.804	1	2.804	22.911	0.000	0.353
Pre.Self-efficacy	7.077	1	7.077	57.834	0.000	0.579
Group	0.128	1	0.128	1.049	0.312	0.024
Error	5.140	42	0.122			
Total	467.208	45				
Corrected total	12.658	44				

## Discussion

The aim of this research was to explore the effects of assisted and unassisted repeated reading on incidental vocabulary learning among Iranian EFL learners. Furthermore, the role of both kinds of the repeated reading in influencing vocabulary learning self-efficacy was examined. The results from this study offer some key findings: First, repeated reading proved to boost vocabulary learning. It was found that both groups (assisted and unassisted) manifested significant improvements in terms of vocabulary learning after the intervention. However, assisted repeated reading helped learners to gain substantially further EFL words. This finding accords with that of Webb and Chang (2012) which revealed that assisted and unassisted repeated reading enhanced vocabulary knowledge of Taiwanese EFL learners with assisted repeated reading contributed to further vocabulary gains. This finding also supports those of Brown et al. (2008) who indicated that reading while listening (assisted repeated reading) is more useful than either listening or reading solely for incidental vocabulary learning. This finding also re-echoes Serrano and Huang’s (2018) claim that assisted repeated reading promotes vocabulary learning in EFL contexts. Since the assisted group used smartphone as the technology device, this finding is also partially in line with previous studies (e.g., Burston, 2013; Rassaei, 2018, 2020; Lin and Lin, 2019) which have emphasized the influence of technology for vocabulary learning.

The findings revealed that vocabulary knowledge was enhanced significantly from multiple exposures to an unknown word – learners seemingly *picked-up* the words. Three interpretations seem valid in this regard. One possible explanation may be that repeated reading coupled with audiotapes provided students with practice in automaticity of vocabulary recognition. Each new encounter with a word during repeated reading might have been conducive to enrich learners’ knowledge of that word through repetition. It can be argued that since the students had more opportunity to re-read the passages, they had greater potential to learn the words incidentally. Put differently, greater opportunity for repetition helped them to consolidate the knowledge of novel and partly known words. This interpretation is supported by some researchers, suggesting that greater frequency of word meeting contributes to word learning (Laufer and Rozovski-Roitblat, 2011).

Second, as the words were met repeatedly, learners could guess their meanings from the context. That is to say, when words are repeated the exposures of the words after the first encounter may provide an opportunity for guessing meanings from the context clues, facilitating retrieval of the meanings of the words obtained from previous meetings. Learners also had the opportunity to learn deliberately through using a dictionary for looking up the meanings of words as there was no time limitation in this procedure.

Third, greater learning may have taken place in the current study due to the fact that the assisted repeated reading group was given the opportunity to read the same passages multiple times while listening to the words being repeated. This suggests that the visual (reading the written passage) and the phonological (listening to the audiotapes) may double the chance of the words being incidentally learned. This means that the passages were more likely to be less effective in enhancing incidental vocabulary learning without re-reading and listening simultaneously. Aural support while reading has been argued to have a positive impact on L2 vocabulary learning (e.g., Brown et al., 2008; Webb and Chang, 2012). It can be claimed that the prosodic characteristics of assisted repeated reading might have aided EFL learners in splitting the linguistic data in chunks more meaningfully, which might have contributed to global understanding of the text. The better global text comprehension, in turn, has enhanced students’ competence in guessing the unfamiliar words.

The second finding of this study was that both kinds of repeated reading contributed to self-efficacy in vocabulary learning among the EFL learners and no significant difference was found between the two types of treatments. Learners’ self-motivated attention to words facilitates their vocabulary learning, which in turn results in greater self-efficacy. Further exposure of both groups to vocabularies included in the short stories might have helped them to enhance their confidence in vocabulary learning. Following Bandura’s (1977) notion of self-efficacy, it can be argued that repeated reading might have boosted learners’ beliefs in their own competencies in directing and executing the learners’ activities and strategies in learning L2 vocabularies. Additionally, since self-efficacy is argued to be correlated with self-regulation (Zimmerman and Martinez-Pons, 1990; Su et al., 2019), it is likely that the two kinds of repeated reading have boosted students’ self-regulation in vocabulary learning, which in turn has contributed to improving students’ vocabulary learning self-efficacy.

Overall, the results obtained from this study provide two findings that inform practice. First, results revealed that both assisted and unassisted repeated reading can be effective approaches for enhancing the amount of L2 incidental vocabulary learning while there can be much to gain from assisted repeated reading procedure which leads to significantly greater vocabulary learning. Repeated reading approach can be used effectively in order to improve learners’ ability to learn vocabulary incidentally. Additionally, both forms of repeated reading increased vocabulary self-efficacy among the EFL learners. The current study findings can expand the existing literature regarding vocabulary learning and repeated reading as it lent empirical support to the usefulness of repeated reading instruction in influencing incidental vocabulary learning.

Assisted repeated reading (in which students listen over and over to an audiotape while reading) helps students comprehend the passage and encourage deeper insights. Additional rereading may help the learners remember more meaningful structures, increase their reading accuracy, and *pick up* the new words. As a result, learners may show greater understanding and use the words mentioned in the text unconsciously while talking about the passage. If a passage is read a number of times, learners benefit substantially by remembering more words.

## Implications

In view of the results of this study, some pedagogical recommendations are suggested to EFL instructors. EFL practitioners are recommended to incorporate smartphones and other applications into their classrooms for vocabulary teaching. As class hours might not suffice for teachers to teach vocabularies effectively, technology devices can help students to learn vocabularies outside the class in their convenient time. Besides, technology devices and applications can not only add fun and excitement to learning but also help EFL students become self-regulated learners (Guirguis and Antigua, 2017). Additionally, practitioners play an influential role in ensuring the usefulness of the repeated reading approach. Therefore, it is needed for future teachers to be trained in executing repeated reading instruction and its techniques such as assessment of learners’ vocabulary level, the selection of appropriate reading materials, and design of reading assignments given to learners. Before implementing assisted or unassisted repeated reading, learners should have a thorough understanding of repeated reading theory, such as benefits, principles, and the ways to make repeated reading successful. Teachers should explain the difference between assisted and unassisted repeated reading and various strategies for developing vocabulary learning before learners start reading.

It is recommended that EFL teachers keep passages short (about 50 to 300 words) because long passages may tire students. They can take various passages from different types of reading materials such as newspaper articles, magazines, novels, and short stories commensurate with learners’ proficiency levels. Reading materials selected for repeated reading program should address learners’ interests and learning needs so as to motivate and energize them to read the passages. It is important not to choose a passage that contains too many unknown words because it might be demanding for learners to comprehend and guess unfamiliar words from the context. If the aim of repeated reading procedure is to enable students to learn words through a particular passage, learners should be given a cue to focus on words and the text should be repeated more than three times.

## Limitations and Suggestions for Further Research

The findings of the current research, nonetheless, have some limitations. To provide a more in-depth and more conclusive evaluation of repeated reading, it is suggested that future studies use qualitative methods in addition to tests or questionnaires in order to present a more detailed and holistic image of the role of repeated reading in improving incidental vocabulary learning. For example, semi-structured interviews about learners’ perceptions towards assisted and unassisted repeated reading would add novel insights into the current literature. Moreover, this study used the data from EFL learners from a private institute in the context of Iran. This context might be radically different compared to other contexts (either private or public schools) in affecting students’ vocabulary learning. Future studies are needed to examine other samples of EFL learners from both the private and public sectors. Furthermore, the existing finding from this sample of Iranian EFL learners may not be transferable to other EFL contexts and cultures. Further research is required to achieve details in association with repeated reading in other populations. In spite of the fact that the outcomes show that repeated reading is useful for learners, the majority of studies have not taken learners’ reading levels into account. Therefore, the success of assisted and unassisted repeated reading for learners with various reading levels cannot be identified. Also, the role of other individual differences in affecting the usefulness of repeated reading should be investigated. Finally, this research did not examine vocabulary retention to find how long such vocabulary learning might last. As a result, it is undefined whether the learned vocabularies were yet retained in the participants’ mental lexicon after the procedure had been finished. Future research thus needs to employ a delayed assessment to investigate the impact of repeated reading on the longer-term retention of acquired vocabularies. Finally, to enrich our understanding regarding repeated reading, exploring the impacts of assisted an unassisted repeated reading on learners’ reading motivation, reading anxiety, and reading attitude would be interesting topics for future studies.

## Data Availability Statement

The raw data supporting the conclusions of this article will be made available by the authors, without undue reservation.

## Ethics Statement

The studies involving human participants were reviewed and approved by University of Kurdistan. The patients/participants provided their written informed consent to participate in this study.

## Author Contributions

HS, FM, and JF were equally involved in designing the research, topic development, data collection, data analysis, writing drafts, and final editing. All authors contributed to the article and approved the submitted version.

## Conflict of Interest

The authors declare that the research was conducted in the absence of any commercial or financial relationships that could be construed as a potential conflict of interest.

## Publisher’s Note

All claims expressed in this article are solely those of the authors and do not necessarily represent those of their affiliated organizations, or those of the publisher, the editors and the reviewers. Any product that may be evaluated in this article, or claim that may be made by its manufacturer, is not guaranteed or endorsed by the publisher.

## References

[ref1] Alahmadi A. Foltz A. (2020). Effects of language skills and strategy use on vocabulary learning through lexical translation and inferencing. J. Psycholinguist. Res. 49, 975–991. doi: 10.1007/s10936-020-09720-9, PMID: 32651841 PMC7661419

[ref2] Anderson N. (1993). “Repeated reading,” in New Ways in Teaching Reading. ed. Day R. R. (Alexandria, VA: Teachers of English to Speakers of Other Languages), 190–191.

[ref3] Bandura A. (1977). Self-efficacy: toward a unifying theory of behavioral change. Psychol. Rev. 84, 191–215. doi: 10.1037/0033-295X.84.2.191, PMID: 847061

[ref4] Bandura A. (1978). Reflections on self-efficacy. Adv. Behav. Res. Ther. 1, 237–269. doi: 10.1016/0146-6402(78)90012-7

[ref5] Bandura A. (2011). On the functional properties of perceived self-efficacy revisited. J. Manag. 38, 9–44. doi: 10.1177/0149206311410606

[ref001] Blum I. H. Koskinen P. S. (1991). Repeated reading: a strategy for enhancing fluency and fostering expertise. Theory Pract. 30, 195–200.

[ref6] Brown R. Waring R. Donkaewbua S. (2008). Incidental vocabulary acquisition from reading, reading-while-listening, and listening to stories. Nat. Foreign Lang. Resour. Center 20:816.

[ref7] Burston J. (2013). Mobile-assisted language learning: a selected annotated bibliography of implementation studies 1994–2012. Lang. Learn. Technol. 17, 157–225.

[ref8] Carrell P. L. Carson J. G. (1997). Extensive and intensive reading in an EAP setting. Engl. Specif. Purp. 16, 47–60. doi: 10.1016/S0889-4906(96)00031-2

[ref9] Carter R. (1987). Vocabulary: Applied Linguistic Perspectives. London: Allen and Unwin.

[ref10] Chomsky C. (1978). “When you still can’t read in third grade. After decoding, what?” in What Research Has to Say about Reading Instruction. ed. Samuels S. (Newark, Del: International Research Association), 13–30.

[ref11] Chou M. H. (2014). Assessing English vocabulary and enhancing young English as a foreign language (EFL) learners’ motivation through games, songs, and stories. Education 42, 284–297. doi: 10.1080/03004279.2012.680899

[ref12] Cronbach L. J. (1942). An analysis of techniques for diagnostic vocabulary testing. J. Educ. Res. 36, 206–217. doi: 10.1080/00220671.1942.10881160

[ref13] Day R. R. Bamford J. Renandya W. A. Jacobs G. M. Yu V. W. S. (1998). Extensive reading in the second language classroom. RELC J. 29, 187–191. doi: 10.1177/003368829802900211

[ref14] Dlugosz D. W. (2000). Rethinking the role of reading in teaching a foreign language to young learners. Eng. Lang. Teach. J. 54, 284–290. doi: 10.1093/elt/54.3.284

[ref15] Ellis R. (2005). Principles of instructed language learning. System 33, 209–224. doi: 10.1016/j.system.2004.12.006

[ref16] ESOL (2009). Cambridge BEC Preliminary: Official Examination Papers from University of Cambridge ESOL Examinations. England: Cambridge University Press.

[ref17] Fathi J. Ahmadnejad M. Yousofi N. (2019). Effects of blog-mediated writing instruction on L2 writing motivation, self-efficacy, and self-regulation: a mixed methods study. J. Res. Appl. Ling. 10, 159–181. doi: 10.22055/RALS.2019.14722

[ref18] Fathi J. Derakhshan A. Torabi S. (2020). The effect of listening strategy instruction on second language listening anxiety and self-efficacy of Iranian EFL learners. SAGE Open 10:878. doi: 10.1177/2158244020933878

[ref19] Fathi J. Greenier V. Derakhshan A. (2021). Self-efficacy, reflection, and burnout among Iranian EFL teachers: the mediating role of emotion regulation. Iranian J. Lang. Teach. Res. 9, 13–37. doi: 10.30466/IJLTR.2021.121043

[ref20] Fathi J. Soleimani H. (2020). The effect of reading strategy instruction on reading self-efficacy and reading attitudes: a case of young female Iranian EFL learners. Appl. Res. Eng. Lang. 9, 382–408. doi: 10.22108/ARE.2019.116944.1461

[ref21] Gorsuch G. Taguchi E. (2010). Developing reading fluency and comprehension using repeated reading: evidence from longitudinal student reports. Lang. Teach. Res. 14, 27–59. doi: 10.1177/1362168809346494

[ref22] Gorsuch G. Taguchi E. Umehara H. (2015). Repeated reading for Japanese language learners: effects on reading speed, comprehension, and comprehension strategies. Read. Matrix 15, 18–44.

[ref23] Grabe W. Stoller F. L. (2002). Teaching and Researching Reading. Harlow, UK: Longman.

[ref24] Guirguis R. Antigua K. C. (2017). DLLs and the development of self-regulation in early childhood. Cogent Educ. 4:1355628. doi: 10.1080/2331186X.2017.1355628

[ref25] Guthrie J. T. Wigfield A. (1999). How motivation fits into a science of reading. Sci. Stud. Read. 3, 199–205. doi: 10.1207/s1532799xssr0303_1

[ref26] Heslin P. A. Klehe U. C. (2006) in Encyclopedia of Industrial/Organizational Psychology. Vol. 2. ed. Rogelberg S. G. (California: SAGE), 705–708.

[ref27] Hong J. C. Hwang M. Y. Tai K. H. Chen Y. L. (2014). Using calibration to enhance students' self-confidence in English vocabulary learning relevant to their judgment of over-confidence and predicted by smartphone self-efficacy and English learning anxiety. Comput. Educ. 72, 313–322. doi: 10.1016/j.compedu.2013.11.011

[ref28] Horst M. (2005). Learning L2 vocabulary through extensive reading: a measurement study. Can. Mod. Lang. Rev. 61, 355–382. doi: 10.3138/cmlr.61.3.355

[ref29] Hunt A. Beglar D. (2005). A framework for developing EFL reading vocabulary. Read. Foreign Lang. 17, 23–59.

[ref30] Islam A. K. M. N. Mavengere N. B. Ahlfors U.-R. Ruohonen M. J. Serenko A. Palvia P. (2018). A stress-strain-outcome model of job satisfaction: the moderating role of professional self-efficacy [Conference Paper]. *24th Americas Conference on Information Systems*, New Orleans, USA.

[ref31] Kuhn M. R. Stahl S. A. (2003). Fluency: a review of developmental and remedial practices. J. Educ. Psychol. 95, 3–21. doi: 10.1037/0022-0663.95.1.3

[ref32] Kweon S. O. Kim H. R. (2008). Beyond raw frequency: incidental vocabulary acquisition in extensive reading. Read. Foreign Lang. 20, 191–215.

[ref33] Laufer B. (2003). Vocabulary acquisition in a second language: do learners really acquire most vocabulary by reading? Canadian Mod. Lang. Rev. 59, 567–587. doi: 10.3138/cmlr.59.4.567

[ref34] Laufer B. Rozovski-Roitblat B. (2011). Incidental vocabulary acquisition: The effects of task type, word occurrence and their combination. Lang. Teach. Res. *15*, 391–411. doi: 10.1177/1362168811412019

[ref35] Lee J. Yoon S. Y. (2017). The effects of repeated reading on reading fluency for students with reading disabilities: a meta-analysis. J. Learn. Disabil. 50, 213–224. doi: 10.1177/0022219415605194, PMID: 26408529

[ref36] Lin J. J. Lin H. (2019). Mobile-assisted ESL/EFL vocabulary learning: a systematic review and meta-analysis. Comput. Assist. Lang. Learn. 32, 878–919. doi: 10.1080/09588221.2018.1541359

[ref37] Linnenbrink E. A. Pintrich P. R. (2003). The role of self-efficacy belief in student engagment and learning in the classroom. Read. Writing Q. 19, 119–137. doi: 10.1080/10573560308223

[ref38] Liu L. Akhter S. Qureshi A. H. (2020). Towards the description of techniques in teaching L2 vocabulary. Revista Argentina de Clínica Psicológica 29:268.

[ref39] Liu Y. T. Todd A. G. (2016). Implementation of assisted repeated reading techniques for the incidental acquisition of novel foreign vocabulary. Lang. Teach. Res. 20, 53–74. doi: 10.1177/1362168814559802

[ref40] Liu H. Wu H. (2011). Impact of extensive reading on young EFL learners’ vocabulary knowledge, reading proficiency, and motivation for learning English. NPUST Human. Soc. Sci. Res. 5, 1–21.

[ref41] Liu J. Zhang J. (2018). The effects of extensive Reading on English vocabulary learning: a meta-analysis. Engl. Lang. Teach. 11, 1–15. doi: 10.5539/elt.v11n6p1

[ref42] Martina F. Syafryadin S. Utama J. A. (2020). The practice of extensive reading among EFL learners in tertiary level. Yavana Bhasha: J. Eng. Lang. Educ. 3, 56–72. doi: 10.25078/yb.v3i2.1712

[ref44] Meyer M. S. Felton R. H. (1999). Repeated reading to enhance fluency: old approaches and new directions. Ann. Dyslexia 49, 283–306. doi: 10.1007/s11881-999-0027-8

[ref45] Mizumoto A. (2012). Exploring the effects of self-efficacy on vocabulary learning strategies. Stud. Self-Access Learn. J. 3, 423–437. doi: 10.37237/030407

[ref46] Mizumoto A. (2013). Effects of self-regulated vocabulary learning process on self-efficacy. Innov. Lang. Learn. Teach. 7, 253–265. doi: 10.1080/17501229.2013.836206

[ref47] Mizumoto A. Takeuchi O. (2009). Examining the effectiveness of explicit instruction of vocabulary learning strategies with Japanese EFL university students. Lang. Teach. Res. 13, 425–449. doi: 10.1177/1362168809341511

[ref48] Nagy W. E. Herman P. A. Anderson R. C. (1985). Learning words from context. Read. Res. Q. 20, 233–253. doi: 10.2307/747758

[ref49] Nakanishi T. (2015). A meta-analysis of extensive reading research. TESOL Q. 49, 6–37. doi: 10.1002/tesq.157

[ref50] Namaziandost E. Alekasir S. Dehkordi E. S. Tilwani S. A. (2021). An account of EFL learners' vocabulary learning in a mobile-assisted language environment: the case of Rosetta stone application. Computer-Assist. Lang. Learn. Elect. J. *22*, 80–110.

[ref51] Nation I. S. P. (2008). Teaching Vocabulary: Strategies and Techniques. Boston: Heinle.

[ref52] Nation I. S. P. (2013). Learning Vocabulary in Another Language. 2nd Edn. (England: Cambridge University Press).

[ref53] Nation I. S. P. Wang K. (1999). Graded readers and vocabulary. Read. Foreign Lang. 12, 355–380.

[ref54] Pavia N. Webb S. Faez F. (2019). Incidental vocabulary learning through listening to songs. Stud. Second. Lang. Acquis. 41, 745–768. doi: 10.1017/S0272263119000020

[ref55] Peters E. Webb S. (2018). Incidental vocabulary acquisition through viewing L2 television and factors that affect learning. Stud. Second. Lang. Acquis. 40, 551–577. doi: 10.1017/S0272263117000407

[ref56] Pigada M. Schmitt N. (2006). Vocabulary acquisition from extensive reading: A case study. Read. Foreign Lang. 18, 1–28.

[ref57] Qian D. D. (1999). Assessing the roles of depth and breadth of vocabulary knowledge in reading comprehension. Canadian Mod. Lang. Rev. 56, 282–308. doi: 10.3138/cmlr.56.2.282

[ref58] Rahimi M. Fathi J. (2021). Exploring the impact of wiki-mediated collaborative writing on EFL students’ writing performance, writing self-regulation, and writing self-efficacy: a mixed methods study. Comput. Assist. Lang. Learn., 1–48. doi: 10.1080/09588221.2021.1888753

[ref59] Ramos R. Dario F. (2015). Incidental vocabulary learning in second language acquisition: A literature review. Profile Issues Teac. Prof. Dev. 17, 157–166. doi: 10.15446/profile.v17n1.43957

[ref60] Rasinski T. V. (1990). Effects of repeated reading and listening-while-reading on reading fluency. J. Educ. Res. 83, 147–151. doi: 10.1080/00220671.1990.10885946

[ref61] Rassaei E. (2017). Effects of three forms of reading-based output activity on L2 vocabulary learning. Lang. Teach. Res. 21, 76–95. doi: 10.1177/1362168815606160

[ref62] Rassaei E. (2018). Computer-mediated textual and audio glosses, perceptual style and L2 vocabulary learning. Lang. Teach. Res. 22, 657–675. doi: 10.1177/1362168817690183

[ref63] Rassaei E. (2020). Effects of mobile-mediated dynamic and nondynamic glosses on L2 vocabulary learning: a sociocultural perspective. Mod. Lang. J. 104, 284–303. doi: 10.1111/modl.12629

[ref64] Read J. (1988). Measuring the vocabulary knowledge of second language learners. RELC J. 19, 12–25. doi: 10.1177/003368828801900202

[ref65] Read J. (2000). Assessing Vocabulary. Cambridge: Cambridge University Press.

[ref66] Richards J. C. (1976). The role of vocabulary teaching. TESOL Q. 10, 77–89. doi: 10.2307/3585941

[ref67] Samuels S. J. (1976). Automatic decoding and reading comprehension. Lang. Arts 53, 323–325.

[ref68] Samuels S. J. (1979). The method of repeated reading. Read. Teach. 32, 403–408.

[ref69] Schmitt N. Jiang X. Grabe W. (2011). The percentage of words known in a text and reading comprehension. Mod. Lang. J. 95, 26–43. doi: 10.1111/j.1540-4781.2011.01146.x

[ref70] Schunk D. H. (2003). Self-efficacy for reading and writing: influence of modeling, goal setting, and self-evaluation. Read. Writing Q. 19, 159–172. doi: 10.1080/10573560308219

[ref71] Serrano R. Huang H. Y. (2018). Learning vocabulary through assisted repeated reading: how much time should there be between repetitions of the same text? TESOL Q. 52, 971–994. doi: 10.1002/tesq.445

[ref72] Shokrpour N. Mirshekari Z. Moslehi S. Popescu M. (2019). Learning vocabulary electronically: does computer assisted language learning (CALL) instruction have any impacts on Iranian EFL learners? Cogent Educ. 6:1702827. doi: 10.1080/2331186X.2019.1702827

[ref73] Song M. (2020). The impacts of extensive Reading on EFL primary school students’ vocabulary acquisition and Reading comprehension. J. Exten. Read. 5, 60–69.

[ref74] Su Y. Li Y. Liang J.-C. Tsai C.-C. (2019). Moving literature circles into wiki-based environment: the role of online self-regulation in EFL learners’ attitude toward collaborative learning. Comput. Assist. Lang. Learn. 32, 556–586. doi: 10.1080/09588221.2018.1527363

[ref75] Suk N. (2017). The effects of extensive reading on reading comprehension, reading rate, and vocabulary acquisition. Read. Res. Q. 52, 73–89. doi: 10.1002/rrq.152

[ref76] Taguchi E. (1997). The effects of repeated readings on the development of lower identification skills of FL readers. Read. Foreign Lang. 11, 97–119.

[ref77] Taguchi E. Takayasu-Maass M. Gorsuch G. J. (2004). Developing reading fluency in EFL: how assisted repeated reading and extensive reading affect fluency development. Read. Foreign Lang. 16, 70–96.

[ref78] Therrien W. J. (2004). Fluency and comprehension gains as a result of repeated reading: a meta-analysis. Remedial Spec. Educ. 25, 252–261. doi: 10.1177/07419325040250040801

[ref79] Therrien W. J. Hughes C. (2008). Comparison of repeated reading and question generation on students’ reading fluency and comprehension. Learn. Disabil. Contemp. J. 6, 1–16.

[ref80] Thornbury S. (2002). How to Teach Vocabulary. Essex: Pearson Education Limited.

[ref81] Wang Y. (2013). Incidental vocabulary learning through extensive reading: A case of lower-level EFL Taiwanese learners. J. Asia TEFL 10, 59–80.

[ref82] Webb S. (2007). Learning word pairs and glossed sentences: the effects of a single context on vocabulary knowledge. Lang. Teach. Res. 11, 63–81. doi: 10.1177/1362168806072463

[ref83] Webb S. Chang A. C. (2012). Vocabulary learning through assisted and unassisted repeated reading. Canadian Mod. Lang. Rev. 68, 267–290. doi: 10.3138/cmlr.1204.1

[ref84] Wigfield A. Guthrie J. T. Tonks S. Perencevich K. C. (2004). Children's motivation for reading: domain specificity and instructional influences. J. Educ. Res. 97, 299–310. doi: 10.3200/JOER.97.6.299-310

[ref85] Yousefi M. H. Biria R. (2018). The effectiveness of L2 vocabulary instruction: a meta-analysis. Asian-Pacific J. Second Foreign Lang. Educ. 3, 1–19. doi: 10.1186/s40862-018-0062-2

[ref86] Zahar R. Cobb T. Spada N. (2001). Acquiring vocabulary through reading: effects of frequency and contextual richness. Canadian Mod. Lang. Rev. 57, 541–572. doi: 10.3138/cmlr.57.4.541

[ref88] Zimmerman B. J. Martinez-Pons M. (1990). Student differences in self-regulated learning: relating grade, sex, and giftedness to self-efficacy and strategy use. J. Educ. Psychol. 82, 51–59. doi: 10.1037/0022-0663.82.1.51

